# MUCOPOLYSACARIDOSIS TYPE IIIB MISDIAGNOSED AS AN AUTISTIC SPECTRUM DISORDER: A CASE REPORT AND LITERATURE REVIEW

**DOI:** 10.1590/1984-0462/2021/39/2019397

**Published:** 2020-10-21

**Authors:** Alan Tibério Dalpiaz Irigonhê, Angélica Malman Thomazine Moreira, Daniel Almeida do Valle, Mara Lúcia Schmitz Ferreira Santos

**Affiliations:** a Universidade Positivo, Curitiba, PR, Brazil.; b Hospital Pequeno Príncipe, Curitiba, PR, Brazil.

**Keywords:** Mucopolysaccharidosis III, Sanfilippo syndrome, Rare diseases, Metabolism, inborn errors, Autism spectrum disorder, Mucopolissacaridose III, Síndrome de Sanfilippo, Doenças raras, Erros inatos do metabolismo, Transtorno do espectro autista

## Abstract

**Objective:**

To report a rare case of mucopolysaccharidosis IIIB in a pediatric patient, with emphasis on the description of the clinical manifestations and the early diagnosis.

**Case description:**

A 14-year-old male patient, who presented regression of neuropsychomotor development since his three years and six months old, with speech loss and frequent falls, evolving with behavioral changes, with agitation and aggressiveness. Although being diagnosed with autism, there was no response to the established treatment; he was subsequently submitted to metabolic investigation, which lead to the diagnosis of Mucopolysaccharidosis IIIB.

**Comments:**

Identifying a metabolic disorder requires connecting multiple signs and symptoms, as well as eliminating other apparent causes. MPS IIIB is a diagnostic challenge, particularly in the early stages and in the absence of a family history of the disease.

## RESUMO

**Objetivo:**

Relatar o caso raro de um paciente pediátrico com mucopolissacaridose III B, com ênfase na descrição de manifestações clínicas.

**Descrição do caso:**

Paciente masculino de 14 anos que, a partir dos 3 anos e 6 meses de idade, apresentou regressão do desenvolvimento neuropsicomotor, com perda da fala e quedas frequentes, evoluindo com alterações comportamentais, agitação e agressividade. Diagnosticado como autista, não obteve resposta ao tratamento estabelecido, sendo posteriormente submetido à investigação metabólica, que evidenciou o diagnóstico de mucopolissacaridose III B.

**Comentários:**

A identificação de um distúrbio metabólico exige conectar vários sinais e sintomas, além de eliminar outras causas aparentes. A mucopolissacaridose III B é um desafio diagnóstico, particularmente nos estágios iniciais e na ausência de história familiar da doença.

## INTRODUCTION

Mucopolysaccharidoses are a group of rare lysosomal storage disorders caused by congenital deficiency of enzymes that catalyze the breakdown of glycosaminoglycans. The lysosomal accumulation of glycosaminoglycan molecules results in dysfunction of cells, tissues, and organs, involving multiple systems, with organomegaly, multiple dysostosis and abnormal facial features.^1^

Mucopolysaccharidosis type III (MPS III), or Sanfilippo syndrome, consists of a disease in which a lysosomal deposit of heparan sulfate occurs. It is caused by dysfunctions in one of the genes that encode the lysosomal enzymes involved in the degradation of this sulfate.^2^ Its prevalence is around 1:200 thousand births and it is among the most frequent mucopolysaccharidoses.^3^ It has four subtypes (A-D), classified according to enzyme deficiency. On the other hand, MPS IIIB occurs due to deficiency of the alpha-N-acetylglucosaminidase enzyme.^4^ Its diagnosis is biochemical, although heparan sulfate can be eliminated in the urine. The definitive diagnosis can be made by proving the low activity of the alpha-N-acetylglucosaminidase enzyme or by detecting pathogenic mutation in homozygosis or heterozygosis composed of the genes involved in MPS III.^4^

It is a progressive disease of three phases, which begin after a period of apparently normal development.^4^ In the first phase, the child has a slight delay in development along with somatic manifestations (recurrence of ear, nose and throat or gastrointestinal diseases). The second phase is characterized by behavioral difficulties, hyperactivity, and sleep disorders. In the last phase, the child suffers loss of intellectual processes and motor functions.^4^ Unlike other mucopolysaccharidoses, which have extensive somatic involvement, patients with MPS III B typically stand out for neurocognitive signs and symptoms.^5^ The condition is predominantly characterized by the presence of severe mental deficiency, neurological degeneration, multiple dysostosis and mild physical problems. Considering it is a rare and little-known pathology, the diagnosis is usually late, which compromises the effectiveness of measures to be taken.^6^

As to autism spectrum disorder (ASD), it manifests as a neurodevelopmental disorder, has an early onset and is characterized by complex behavioral deficits, including language disorders, impaired social interactions and restrictive, repetitive and stereotyped behaviors.^7^ ^,^ ^8^ The prevalence is 6:1,000 and is more common in men than in women.^8^ The etiology of ASD is unclear and more than 100 genes and 44 genomic loci are involved in its pathogenesis.^7^ Genetic factors play an important role in the development of this condition, seen that the heritability rate of the disorder is approximately 80%.^9^ The American Academy of Pediatrics guides targeted research in case of suspicion of specific genetic disease and, in other situations, analysis of chromosomal microarray, research of fragile X in families with an inheritance pattern linked to sex and suggests considering the sequencing of the gene MECP2 in girls. In cases in which the investigation is inconclusive, complete exome sequencing can be performed.^10^

The present case report aims to illustrate the importance of the etiological diagnosis of a patient diagnosed with ASD and to alert to the suspicion of MPS III.

## CASE REPORT

A 14-year-old male patient, referred to a referral hospital service in Curitiba City, Paraná State, Brazil, for investigation. He was born at 38 weeks, weighing 3,500 g, measuring 52 cm, and head circumference not informed. Mother denied consanguinity. The child sustained his head at 4 months old, sat without support at 6, crawled at 9, walked at 1 year and 4 months old and spoke at 2 years old. Until then, the patient had an appropriate development for his age.

The earliest sign of developmental change was speech regression, which started after 2 years of age. Speech was completely lost at 3 years and 6 months old. During this period, frequent falls occurred, and the worsening of balance was progressive. The loss of balance became more constant, making mobility very difficult.

Subsequently, the patient presented psychomotor agitation, hyperactivity, insomnia, and loss of urinary and anal sphincter control. He evolved with a progressive worsening of behavior, with crises of psychomotor agitation increasingly serious and frequent, accompanied by obstinacy, periods of aggression, restlessness and a rapid decrease in attentional capacity. It demonstrated stereotypes, such as cutting paper and hitting cans over and over again. At 7 years of age, he presented coprophagia and spread feces on his own body. He was diagnosed with ASD at the age of 6 and, at the age of 8, he started to be monitored in a special school. He was administered risperidone, amitriptyline, clonazepam, fluoxetine and periciazine, but with no adequate response. At the special school, he attended occupational therapy and speech therapy, both of which were unsuccessful.

At the beginning of the case, despite the falls, he was able to walk with the aid of crutches, but at 12 years and 11 months old he suffered a complete loss of function. There was also a decrease in his swallowing capacity, until the point in which he needed assistance from a nasogastric tube for nutrition. At the age of 13, gastrostomy was inserted to give him food. As his condition evolved, the behaviors of hyperactivity and aggressiveness decreased, with a gradual and progressive transition to drowsiness since the age of 9, and the intense drowsiness occurred around 12 years of age.

In the previous morbid history, there are reports of recurrent respiratory tract infections. However, there is no history of fainting or seizures, nor a family history of genetic, neurological, psychiatric or cardiovascular diseases.

On the physical examination, his weight was 25 kg (Z score: -3.81), his height, 125 cm (Z score: -4.05), body mass index of 16 (Z score: -1.26) and head circumference of 53 cm (Z score: -1.33); coarse facial features, saddle nose, thick lips, sinophris, short neck, macroglossia, prominent forehead, dry and thinning hair; and osteotendinous reflexes 3+/4+, claw hands, spasticity in lower limbs and joint stiffness. Ophthalmological examination as normal, extrinsic eye movement apparently preserved, with bilateral positive red reflex, fundoscopy/retinal mapping without abnormalities.

Magnetic resonance imaging of the skull showed slight dilation of the lateral ventricles and the third ventricle, with diffuse thickening of the diploe and volumetric decrease in the cerebral hemispheres. Magnetic resonance imaging of the total spine showed an inversion of the physiological cervical curvature, diffuse morphostructural alteration of the vertebral bodies and wedging of their plateaus, promoting the radiological aspect of the bullet-shaped vertebra. Diffuse disc dehydration associated with a slight reduction in intervertebral spaces and small intrasomatic disc herniations (Schmorl’s nodules) of chronic aspect in the contiguous plateaus.

The echocardiogram identified hypertrophic cardiomyopathy, with significant left ventricular hypertrophy and moderate diastolic dysfunction, mild double aortic lesion and moderate aortic insufficiency. The electrocardiogram showed left ventricular overload. Abdominal ultrasound revealed liver with normal contours, regular echogenicity, and echotexture, but enlarged dimensions.

The patient had increased urinary glycosaminoglycan excretion, with elimination of heparan sulfate. Laboratory tests showed glycosaminoglycans=127 µg/mg creatinine (reference value 44-106). The diagnosis was confirmed by evidence of deficiency of the alpha-N-acetylglucosaminidase enzyme. Laboratory tests showed:

- N-acetyl-glucosaminidase dosage: not detected (reference value=10-34).
- Acetyl-CoA glucosamine-N-acetyltransferase: 11 nmol/17 h/mg protein (reference value=14-81).

## DISCUSSION

MPS III presents its first symptoms usually between 1 and 3 years of age, in the so-called first phase, with the slowdown or stabilization of cognitive development.^3^ Somatic manifestations related to the upper respiratory tract and the gastrointestinal tract can also occur.^4^ Speech is the most visibly affected of the functions. In a large French study with 107 patients, speech delay at diagnosis was observed in 92% of patients with MPS IIIB. Motor development in general progresses normally during this stage.^3^ In the present case, the patient had earlier speech disorders, which culminated in his total loss at 3 years and 6 months old, an age compatible with that found in the literature. It is also possible to observe a history of respiratory infections, which can be the initial somatic manifestations of the disease.

The second phase of the disease begins between 3 and 4 years old, characterized by progressive cognitive deterioration, and the appearance of behavioral difficulties and sleep disorders. Hyperactivity, impulsiveness, obstinacy, anxious and autistic behaviors worsen over time and can become extreme, to the point that parents report the need for constant supervision. Impulsivity can be such that patients act without showing concern for their own safety.

In the second phase of disease, the child in the reported case presented behaviors like those described in the literature. Another coincident factor of this case with the reports in the literature was the change in the sleep pattern: more than 90% of these children have sleep disorders, which can be debilitating for the family. Patients remain active throughout the night: they walk, sing, scream or speak, and fall asleep for just a few hours.^5^ In this phase, children can be easily misdiagnosed with ASD or developmental disorder, because this is when social reciprocity and communication are compromised. However, these children have no restricted interest or repetitive behavior.^11^ With the incorrect diagnosis, they can suffer unnecessary or harmful approaches, like what happened to the child of the present report. The child in question received a diagnosis of ASD and started treatment for this disorder, with follow-up in a special school, use of indicated medications and occupational therapy, in addition to speech therapy, without success. In such cases, it is frequent for patients not to respond or respond poorly to standard medications and behavior-based interventions.^5^ The correct and early diagnosis of MPS III allows for an equally early intervention, which includes genetic counseling for the family, greater eligibility for effective treatments and, potentially, better quality of life, because with the correct diagnosis, it is possible to offer personalized behavioral support, including interventions that support communication and social skills.^12^

Finally, the third phase usually begins in adolescence, with marked severe dementia and a decline in motor function. In this phase, somatic symptoms stand out and behavioral problems slowly decrease as patients lose functional independence. Patients eventually regress, until they become completely bedridden, in a vegetative state, and have difficulties in swallowing and spasticity. They usually die at the end of the second or at the beginning of the third decade of life.^5^ In this phase, the patient described decreased hyperactivity and aggression, moving to a state of drowsiness and apathy, as described in the literature.

**Figure 1:**
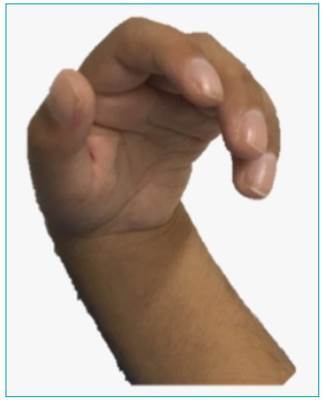
Phenotypic characteristics of the syndrome: trigger fingers.

**Figure 2:**
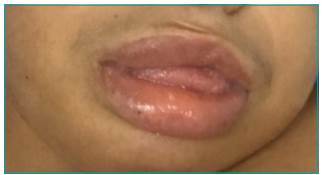
Phenotypic characteristics of the syndrome: lingual protrusion and thick lips

Regarding the examination findings in the case reported, characteristic changes of MPS IIIB were observed, such as: orthopedic changes (Figure 1), facial changes (Figure 2), recurrent respiratory infections, cardiac changes, and hepatomegaly. There are reports in the literature that such orthopedic manifestations (scoliosis, kyphosis, lumbar lordosis, dysplasia and hip pain, carpal tunnel syndrome and trigger fingers) are a characteristic of MPS IIIB, although they are late and not so frequent. Other common signs of MPS IIIB include mild facial dysmorphisms, frequent ear or respiratory tract infections, valvular heart disease, hernia and hepatomegaly,^5^ findings found in the patient in question.

The biochemical diagnosis of mucopolysaccharidosis begins with the quantification of excretion of glycosaminoglycans in the urine, which must be associated to the clinical manifestation of the patient.^13^ In laboratory tests prior to referral, the accumulation of heparan sulfate in the urine was observed, suggesting mucopolysaccharidosis.

There is no doubt that MPS IIIB is a diagnostic challenge, particularly in the early stages and in the absence of a family history of the disease, especially in our country, where information on innate metabolism errors is still limited. The identification of a metabolic disease requires connecting several signs and symptoms and eliminating other apparent causes. The somatic manifestations are heterogeneous and can be much more subtle than those observed in other disorders of mucopolysaccharidosis. In addition, some patients have delayed global development, which can lead to diagnostic confusion.

In daily practice, it can be a diagnostic challenge for the clinical pediatrician to distinguish the behavioral difficulties of ASD from MPS IIIB, particularly in the early stages. It is important to review the milestones of child development, as well as to request additional tests in the face of a case that raises doubts. The progressive loss of childhood neurodevelopmental milestones can be a warning sign for differential diagnoses to be investigated. When there is diagnostic doubt, the possibility of an innate metabolism error should be considered. This attitude allows early diagnosis with the intent of optimizing treatment and avoiding exposure to inappropriate drugs and therapies.
